# Using digital health to enable ethical health research in conflict and other humanitarian settings

**DOI:** 10.1186/s13031-018-0163-z

**Published:** 2018-05-14

**Authors:** Eric D. Perakslis

**Affiliations:** 1 Datavant Inc., San Francisco, CA USA; 2 Department of Biomedical Informatics, Harvard Medical School, Boston, MA USA; 3 Manson Unit, Médecins Sans Frontières, London, UK

**Keywords:** Digital health, Humanitarian research, eHealth, mHealth, Data privacy, Data security, Data sharing, Biometric identity, Research ethics

## Abstract

Conducting research in a humanitarian setting requires quantifiable quality measures to ensure ethical study conduct. Digital health technologies are proven to improve research study quality and efficacy via automated data collection, improvement of data reliability, fidelity and resilience and by improved data provenance and traceability. Additionally, digital health methodologies can improve patient identity, patient privacy, study transparency, data sharing, competent informed consent, and the confidentiality and security of humanitarian operations. It can seem counterintuitive to press forward aggressively with digital technologies at a time of heightened population vulnerability and cyber security concerns, but new approaches are essential to meet the rapidly increasing demands of humanitarian research. In this paper we present the case for the digital modernization of humanitarian research in conflict and other humanitarian settings as a vehicle for improved research quality and ethics.

## Background

While there is justifiable concern, dialogue and debate on the necessity of research in conflict and humanitarian settings, research is being conducted and likely will continue to be conducted in these settings. Governments, non-governmental organizations (NGOs) and the international community must understand the benefit-risk ratios and efficacy of interventions, and the resulting health, social and economic outcomes following such interventions. Concurrently, it must be clear that the rights, confidentiality and identity of all research subjects are protected and that all possible harms were minimized [1]. Researchers are consistently studying and reviewing these and other ethical obligations, and there has been substantial progress in developing methods and practices to ensure ethical research conduct in humanitarian settings [2, 3]. Despite this progress, questions remain. Is all ‘interesting’ research necessary? Does the production of high quality evidence hinder the ability to provide the most effective interventions possible to the most vulnerable populations [4]? These questions are difficult and necessary, as consideration of research must always include definitions and measures of benefit-risk ratios and proper ethical oversight before, during, and after humanitarian interventions. This writing proposes that modern digital technologies can improve the ethics and benefits while reducing the risks of research conduct in humanitarian settings.

In recent reviews on health and humanitarian crisis, two of the primary recommendations were the “ethical imperative” of collecting better data and the need for better information systems [5, 6]. Better data is: generated by valid experimental designs; timely; statistically rigorous; properly protected; useful for local authorities; obtained only through proper (truly) informed consent; and capable of providing an evidence base to support the resulting conclusions and recommendations of a given study. Better information systems are: international standards-based; available but secure; quickly available when crises occur; interconnected; cost-effective; and operationally accessible and useful to local authorities. Ideally, better systems can also be an important component in strengthening local health systems as was shown during the recent Ebola epidemic in West Africa [7]. Despite the incessant marketing hype, digital health technologies are designed to improve data quality and security, systems availability and systems flexibility. In addition, digital systems have great potential in patient/subject identity tracking and identity protection, improved communications, data confidentiality, data redundancy, data protection and local workforce development [8].

## Overview of the utility of *e*Health and *m*Health in humanitarian research settings

The recent Ebola crisis in West Africa highlighted the limitations of paper systems for patient care, research and logistical support during an infectious disease outbreak. While common and readily available, paper is fragile, easily damaged by weather, greatly limited by the skills/literacy of the user, often difficult to read or reproduce. Most of all, paper itself can be a transmission vector during infectious disease epidemics [9]. Digital technologies, on the other hand, continue to improve and to be proven effective, even in low resource settings when properly engineered and implemented. Success is far from automatic. Essential elements for successful digital technology implementation include proper methodologies, qualified personnel, strong use case and scenario selection, realistic expectations and high-touch stakeholder management [10]. Unless these elements are fully understood and effectively executed, digital technology implementation can result in costly mistakes. The fact that massive amounts of resources continue to pour into digital technologies should provide optimism that these solutions are in close reach and will continue to improve.

One important challenge of conducting research in conflict and humanitarian settings is that it is resource-intensive and could divert attention from patient care [11]. In some cases, this resource burden can be offset by automation. When data is captured electronically, some tasks are automated, and others can be expedited. Conduct of surveys provide the most common example. Paper surveys can be time consuming, illegible, poorly understood by the worker administering the survey, easily damaged by weather or transport and easily stolen or destroyed by hostile actors. The author witnessed this first-hand when deploying a community surveillance mobile app in Kono Sierra Leone during the recent Ebola epidemic. Not only was the app found to be quickly superior to paper for data collection, data quality, data protection and accuracy, the program has proven to be useful for healthcare systems strengthening as there are now more than 100 community health workers using the system [12]. In addition to disease epidemic information, clinical knowledge inside conflict settings has also been successful collected through electronic surveys. One recent study polled healthcare providers within Syria to test awareness of tele-mental health (TMH). The study showed that initial awareness of TMH was low but that the polled physicians were interested, willing to try and thought that such interventions could be effective [13]. Indeed, these tools can actually reach massive numbers of users and bring great utility in a short period of time. This was the case with a Médecins Sans Frontières’ Clinical Guidance mobile application which was downloaded in 150 countries and resulted in 250,000 screen views in the first 6 months [14]. These are just a few examples, but the literature and the technology press are blooming with new examples monthly, and best practices are rapidly emerging.

The arguments against the use of digital health tools for the purpose of expanding the reach and minimizing the resource burden of research is that the technology will be too foreign, too complex and too difficult for successful utilization within some settings. While these concerns are valid and important, the landscape is evolving very quickly. Digital transformation appears to be accelerating in low resource areas and conflict zones. One fascinating example is the rapid adoption of cashless currency in challenging settings such as Somaliland. Indeed, even in a country with very high illiteracy rates, it is both simplicity and enhanced functionality that are helping cashless currency flourish [15]. Clearly, the familiarity of cellular phones and tablets is on the rise in low and middle-income countries (LMIC), and this trend will help offset the concern that these technologies appear too foreign.

The potential complexity and difficulty of using digital health technologies must be managed and mitigated carefully by experienced personnel. Digital projects fail primarily due to project management and social issues, regardless of country income level [16]. The most common reasons for failure include avoidance of root cause challenges, unclear or under-articulated goals, lack of proper methodology, lack of understanding of true customer needs, inadequately qualified leadership and staff, poor technology selection, poor communication and poor change management. While information technology (IT) project management is beyond the scope of this writing, Table 1 shows common technology delivery project pitfalls and offers practical guidance [17–22].

**Table 1:** Best Practices and Common Mistakes in Digital Healthcare Implementation

Challenges in *e*Health & *m*Health Delivery	Best Practices & Specific Methodologies
Poor or limited user involvement & engagement	User-centered design, user co-design, participatory design methodologies
Unclear goals, expectations & scope creep	Develop & use a clear requirements & expectations matrix
Poor sponsor participation & active leadership	Document specific sponsor role requirements & the corresponding relationships to other roles
Poor technology selection	Use an established technology selection framework
Lack of necessary technology skill sets	Understand the necessary roles & recruit, train or contract
Poor project management & lack of formal methodology	Understand & select from 6 most common technology delivery methodologies

## Ethical issues of Health Research to be addressed

The basic principles behind ethical human subject research are well articulated and include respect for persons (and their choices), beneficence (the research must do good), non-maleficence (the research does not harm) and justice (all persons are treated fairly and equally) [23]. These principles are elaborated on within the International Ethical Guidelines for Health-Related Research Involving Humans published by the Council for International Organizations of Medical Sciences (CIOMS) in collaboration with the World Health Organization (WHO) [24]. Despite these guidelines, the application of ethical frameworks to digital health is still new.

It is not always clear how to best apply specific ethical guidelines to new technologies. Technology can be unfamiliar, scary and intimidating. Common concerns include training, accuracy, reliability, privacy, security, inequality and protection of relationships [25]. Despite CIOMS guidance, further subtleties that must be examined are the differences in the collection and use of aggregate population data versus individual patient data. One emergent example is the debate around the use of aggregate phone call detail record data from mobile phone systems within low and middle-income countries (LMIC) [26]. When aggregate data is being made available for research via third parties, how is consent handled? Is the research really in the best interest of the consumers about whom the data was collected? One of the primary requirements of research, of course, is trust between the various actors. However, in fragile states and during conflict, this trust is often missing which can greatly inhibit participation [27]. When considered thoroughly, these concerns are not new, unique or limited to conflict and humanitarian settings. In fact, these concerns are the same as are being actively debated and managed in most healthcare systems, but additional protections must be enabled for the most vulnerable people.

Some argue that the only answer to the collective challenges of dire unmet humanitarian need and significant ethical hurdles is the forward press of innovation. Indeed, Médecins Sans Frontières considers innovation an essential element of humanitarian response and has published a framework for humanitarian innovation that considers harms, benefits, local participation, longer-term consequences and specific delivery methodologies [28]. Similarly, it has recently been suggested that, with proper education and outreach, *m*Health and telehealth offer a relatively low-resource platform for the Sustainable Development Goal (SDG) 3 in conflict-affected populations [29]. There has also been excellent recent work done to assess and describe responsible data approaches for humanitarian settings. Specifics include risk assessment, data value chain, legal foundations, and accountability and best practices [30].

Using the growing body of positive evidence of digital capabilities, an association can be constructed between specific CIOMS guidelines and the best practices of digital technologies as shown in Table 2. Each digital capability enhancement opportunity will be discussed in detail.

**Table 2:** Association of Specific CIOMS Guidelines and Digital Technology Improvement Opportunities

International Ethical Guideline (CIOMS)	Digital Enhancement Opportunities
Informed Consent (Guidelines 9,10,16)	Better comprehension via multi-media, improved privacy, traceability (including ability for withdrawal) & confidentiality
Collection Storage & Use of Data (Guidelines 11&12)	Improved Data Quality, Fidelity, Provenance, Data Reliability
Privacy and Confidentiality (Guidelines 3,4,11,12,20,22)	Digital Identity. Data Resilience, Data Redundancy
Data Transparency & Sharing (Guideline 8,12,22)	Increased Data and Study Transparency

## Technologies for improved ethical informed consent

Properly documented informed consent is an essential basis of ethical human subject research. All studies are ethically and legally bound to ensure that any and all potential research participants fully understand all aspects of the process they are being asked to undertake. This requires that potential research subjects receive, comprehend and make decisions on information that can be completely beyond experience or understanding. Common challenges include basic literacy, health literacy, the proper local context, cultural competency, proper documentation and the challenge of successfully communicating complex research and clinical protocols [31, 32].

These challenges are not limited to humanitarian or low-resource settings, as the entire world struggles to ensure that the informed consent process truly satisfies its ethical purpose and study documentation purposes [33, 34]. Fortunately, progress is being made, and technology is playing a greater role. For example, digital informed consent tools can include multi-media videos, stories, pop-up definitions and quizzes, all of which have been shown to improve patient comprehension and retention [35]. But are the same principles and tools being used to improve the informed consent process in the industrialized world suitable to humanitarian use? Early evidence is positive, but challenges remain. Multiple studies of informed consent across multiple medical discipline and in various developing nations show that the use of audio and visual multimedia demonstrate quantifiable improvements in understanding and retention [36]. The challenges reported include fear of data and privacy concerns and hesitance by potential research subjects to sign off on the consent forms.

With respect to data and privacy concerns, one of the risks is that more data can be collected than a subject understands. For example, apps could passively capture the GPS coordinates of the exact location of the consent, and this data could be used by other parties if the devices were not adequately protected and controlled [37]. This may be difficult, or impossible, to effectively communicate. With respect to the specific concern regarding signatures, a recent study in northern Ethiopia found that subjects were afraid to sign consent forms due to lack of trust of investigators and the concern that signatures could be related to legal accountability [38]. Clearly, there are important patient sensitivities and concerns regarding privacy, the potential misuse of personal information and fear of unintended consequences. This is where digitized personal identity may help greatly.

## Next-generation digital identity and identity/privacy protection

The United Nations Sustainable Goal 16.9 calls for legal identity for all citizens including birth registration by 2030 [39]. This goal is aspirational and complex as no truly ideal global identification strategy exists. The complications of an unprecedented refugee crisis, unstable states and exploding identity theft and misuse in the industrialized world make this a global problem for all peoples, not just those in developing nations. The particular challenges to uniform global identity solutions include the lack of consistent state-issued identification (ID), political instability, corruption and fear of persecution and stigma. Indeed, even in the most developed nations, there is a growing trend of individuals that are choosing to live off-the-grid [40]. For many peoples, feeling safe has a great deal to do with feeling anonymous.

But what aspects of life need to be associated with identity? In the US, past and present identity schemes involve personal information that are based around establishing uniqueness. Date of birth, place of birth, social security number and other personally-identifiable-information (PII) such as home address form the basis of modern identity and, truly, most of this information has likely already been stolen. Estimates vary but the data breaches are now affecting 100 s of millions of citizens per year worldwide. From the healthcare perspective, in the US, personal health information (PHI) is further protected by the Health Insurance Portability and Accountability Act (HIPAA), although here, too, cyberattacks are all too common as this data is considered highly valuable [41, 42]. Many now question this strategy identifying people with personal information and then de-identifying those same people as research subjects by stripping a subset of the personal data. Must unique identification be based on some of the most personal and private attributes of life? Probably not, as newer technologies and smarter identity schemes are rapidly evolving.

Digital identity is likely the best path forward given the complexities and the financial, health and security issues around global identity [43]. Ideal solutions must be truly unique (at least nationally), portable, resilient, inexpensive and standards-based to allow interoperability with national systems. For populations at risk or threat, the additional capability to support those living on-the-grid and off-the-grid will be essential. This can be less tricky than it seems. When unique identity schemes do not depend on associated personally-identifiable data, the risk to individuals is greatly decreased. One recent article suggested that there ‘should be an outcry to eliminate the brandishing of birthdates to identify patients in medical encounters’ [44].

Fortunately, in the case of biometric ID, the source of uniqueness is not marketable information such as PII or PHI; it is simply biological traits, such as fingerprint or retinal scan, which need not be associated with any personal information to be fully unique [45]. In many ways, this strategy is essentially proactive de-identification according to HIPAA guidelines as long as none of the 18 types of identifiers are ever associated with the ID [46]. These technologies are rapidly evolving. In fact, in 2016, new national electronic ID (*e*ID) programs, most including biometrics, were announced in Algeria, Cameroon, Jordan, Italy, Senegal and Thailand, and pilots were launched in many other nations [47].

Also driving progress are federal government guidelines such as the US NIST SP 800–63 Digital Identity Guidelines. These guidelines provide comprehensive guidance on digital identity, enrollment, identity proofing, authentication and lifecycle management [48]. While not yet mandated, it is expected that these guidelines and equivalents from other nations will set the bar for quality and responsibility of national ID systems and must be carefully considered.

Admittedly, this is a great deal of technology and complexity to comprehend, and many humanitarian missions and settings lack the required technological sophistication to do so. This is where digital identity services can bring excellent value. Digital identity services provide identity solutions ‘as a service’ and are now being used by many sectors worldwide. The growth of this industry is so explosive that the greatest challenge can be selecting the optimal solution for a given purpose. Here again, there are excellent international guidance documents available to inform and guide [49].

## Improving data reliability, fidelity and resilience

For data to be reliable it must be accurate, precise and available. Each of these can be aided by digital tools. For example, digital surveys greatly improve data accuracy and fidelity by enforcing data types such as numerical fields, date fields etc. that ensure proper answers. Multiple choice questions ensure specificity, precision, legibility and suitability of answers. Data availability can be improved by local data caching on devices as well as downloading copies of data via wireless networks (Wi-Fi) or to other devices via peer-to-peer data transfer. Data redundancy is also the best protection against data loss in any setting.

Data resilience is the ability to recover from loss or incident, and this is where digital methods greatly improve upon most paper systems. Paper can get wet or lost, or simply be illegible by the time of intended use. Digital devices offer instantaneous redundancy, even where there is no cellular or Wi-Fi capability, and can store and share copies while offline. Paper also becomes onerous to store and archive. Large missions can quickly compile stacks, boxes and rooms of poorly annotated and filed data making re-use and long-term utility difficult.

## Improving data provenance

Another essential element of well-conducted ethical research is proper data provenance. Data provenance is the ability to describe the history and origins of data, a critical element of data reproducibility. The ability of digital systems to create metadata that can be used to establish and ensure data origin, chain of custody and reproducibility is a significant improvement over traditional paper procedures. These improvements can be further enhanced by many pre-existing ontologies that enable the use of data standards and the ability to automate data integrity checking [50, 51]. Lastly, while much of the data provenance and provenance metadata literature is focused upon highly technical and advanced cloud computing environments, it is essential to understand that the concepts are fully amenable to much lower tech environments. Solid experimental data provenance can be established using techniques such as basic labeling and tracking, using proper version control and backups, smart use of data identifiers and even hybrid digital and paper processes [52].

## Data quality, data protection and research cyber security

The author has previously provided guidance for research study cyber security and privacy protection so will not dive deeply into these technicalities in this writing [53]. The most important aspects to consider in humanitarian settings are the specificity of the environment, prioritization of data and systems, access and identity management, proper device patching and management, comprehensive daily data backups, good physical security and regular testing of all procedures and technology controls [54].

Fundamental to all security and privacy strategies is an understanding that all data is not of equal risk and importance. In the wrong hands, a clinical case report form that identifies a subject solely based on a unique patient ID, carries much less risk to the patient and/or provider than does the spreadsheet or database that associates personal information with those unique patient IDs.

Data is not of equal risk and this is the basis behind HIPAA, GDPR and other privacy laws. These regulations must be seen as an opportunity to make research more efficient, portable and transparent. Decide what is important and protect what is important. Worry much less about everything else.

In considering data privacy and utility, electronic data can be more useful and secure overall. Consider the case of collecting and managing informed consent forms during any large medical intervention or study. If paper forms and wet signatures are used, what are the odds that a subject could be found and competently re-identified in a crowd fifteen minutes later? Chain of custody of data, including the ability to attach results and documents to particular subjects, is fundamental to ensuring study quality. Now consider the same scenario where an electronic case report form app and a digital biometric identity were used; instant and highly reliable re-contact would be possible and credible as needed.

One last important topic on cyber security is a specific caution around the Android operating system. The Android operating system is far more ‘open’ than the analogous iOS operating system used by Apple. This has truly enabled rapid and worldwide utility of mobile applications. Most open-source software systems run Android and most reasonably priced phones and tablets run Android. Because Apple controls the entire iOS ecosystem, Apple devices tend to be more secure. They should be, given the $1000 price of the new iPhone! Practicality and economics will cause most of the work to remain on Android, and this is okay, as long as users are vigilant. Technology strategies that rely on Android OS, especially those that handle sensitive information, must be carefully managed. Android devices can indeed be as secure as iOS devices if managed correctly [55].

## Pitfalls of digital data management in research settings

In addition to the previous cautions on education and training, project management, proper sponsorship and staff involvement, there are specific cautions that must be understood when implementing digital data collection technologies. First there are the logistical requirements of managing devices, managing users and protecting against theft and misuse. Next are the operational and technical requirements of ensuring that devices can be properly charged, cleaned and kept in good working order. In highly challenging physical environments that may be wet, dry or dusty, proper protective casings and an adequate store of spare devices is required. Lastly, it is undeniable that these technologies and the corresponding preparation and management add financial cost to any research study, but the return on these investments can be extraordinary.

## Data and study transparency

It has been argued that the attainable minimal quality standard in epidemiology is reproducibility, and that availability of data sets, software, detailed protocols and statistical approaches enables the types of critical evaluation that ensure study quality and transparency [56]. Maximum transparency is considered an essential element of ethical research as it ensures people are treated properly and that the research itself was conducted with the best interests of the most vulnerable in mind [57]. For industry sponsored clinical trials, transparency via access to data, protocols and results is expected and mandated, although performance varies greatly [58]. While there is clearly much room for improvement in the way that industry shares clinical trial data, the fact is that industry does systematically share data, and there are no truly comparable sharing efforts within academia or the humanitarian sector. Commitment to open sharing of study data would truly raise all boats with respect to the perceptions and concerns regarding the ethics of conducting research in humanitarian settings, and technology can only help.

Data that has been systematically collected, properly managed and evaluated using rigorous statistical methods can be readily examined and evaluated by editors, reviewers and other researchers. Studies that lack well-controlled source data have inadequate chain of custody and lack procedural rigor account for a great proportion of irreproducible research. In contrast, simple checklists have been shown to improve methodological information such as randomization, sample-size calculation and blinding [59]. As previously mentioned, checklists can be readily automated via digital means and can even be improved upon as data quality and completeness can be managed as mandatory.

In addition to ensuring quality, digitally shared data can be aggregated, aligned and pooled or even co-located to produce rich new sources of knowledge. A common driver of these efforts is to facilitate knowledge sharing in hopes of preventing future humanitarian crisis. One such effort is underway to pool data from the recent Ebola outbreak in West Africa. The issues of data ownership, control and access all must be settled [60].

## Conclusions

While digital health is imperfect and still in its adolescence, the field is rapidly evolving. New digital studies and capabilities are being reported almost daily, and many have the ability to improve the ethical conduct of research in humanitarian settings. By automating chain of custody of data, by using smart metadata and by exploiting the other inherent capabilities of digital technologies, the quality and conduct of research in humanitarian settings can improve. The change will not be easy, but the rewards appear worth the risk.

The decision to conduct research in humanitarian settings is incredibly complex, and a case can often be made against intervention. However, when the decision to intervene is made, that intervention must be thorough and profound, as each clinical interaction happens only once and is irreplaceable.

## Abbreviations

*e*Health: Electronic health
*e*ID: Electronic ID
HIPAA: Health insurance portability and accountability act
ID: Identification
IOS: Apple proprietary operating system
*m*Heath: Mobile health
NGO: Non-governmental organizations
PHI: Personal health information
PII: Personally-identifiable information
TMH: Tele-mental health
WiFi: Wireless network
